# Reviewers acknowledgement

**DOI:** 10.1002/2211-5463.12546

**Published:** 2018-12-02

The Editors of *FEBS Open Bio* would like to thank all those who have given their time and expertise to review articles submitted for publication in Volume 8. Although *FEBS Open Bio* does not seek to judge the importance of submissions, our reviewers carefully scrutinize the experimental design and results of all papers, and challenge authors about their conclusions.

The names of these reviewers are listed below; we apologize if we have inadvertently omitted anyone.

Michael Acker

Luis Alberto Acuña‐Amador

Itsuki Ajioka

Yusuf Akhter

Salam Al‐Karadaghi

M R K Alley

Peter Alston

Luis M Alvarez‐Salas

Zandrea Ambrose

Jesus Angulo

Yutaka Aoyagi

Elisa Araldi

Tamas Aranyi

Paolo Arosio

Manuel Aviles

Julio Ayala

Michelle Baack

Sylvie Babajko

Anand Kumar Bachhawat

Marcio Bajgelman

Abhijeet Bakre

Hanna‐Mari Baldauf

Clair Baldock

Ryoichi Banno

Zhao‐shi Bao

Cristina Barbagallo

Marco Baroni

Eliaz Barquero

Reetobrata Basu

A Hilal Bati

Oliver Batistic

Giorgia Beffagna

Gusztav Belteki

Sam Benchimol

Iddo Ben‐Dov

Alexandre Benedetto

Simone Beninati

Rita Bernhardt

Anthony C Bishop

Andrew Blackford

Carl P Blobel

Marie Boudsocq

Marie‐Agnes Bringer

Bernhard Brüne

Thomas Brunner

Bernd Bukau

Brant Burkhardt

Peter Burkovics

Joel N Buxbaum

Jonas Bystrom

Lisa D Cabrita

Carme Caelles

Jinquan Cai

Mariá Calzada

Teresa Cardoso

Adriana Karaoglanovic Carmona

Sebastien Carpentier

Maria Teresa Carri

Fabio Cattaneo

Eduardo A Ceccarelli

Gianna Cecchetto

Valentina Celestini

Pimchai Chaiyen

Sajal Chakraborti

Roger Chammas

Dedrick Chan

Eric Chang

Max Chavarria

G Chen

Dongdong Cheng

Peter Cheung

Fulvio Chiacchiera

Christine Chio

Kamal Chowdhury

Ping Chung Leung

Gabriela Ciapetti

Elisa Cimetta

W H Colledge

Gerolama Condorelli

Ivan Conte

Angel Corbi

Jose Correa

Francisco Corzana

Jean‐Francois Cote

Peggy A Cotter

Danilo de Menezes Daloso

Jiří Danihlík

Padraig D'Arcy

Anibh Das

Xavier Daura

Marie Davídková

Keith R Davis

Walter De Azevedo

Alfredo De Biasio

Xavier De Bolle

Bart De Geest

Gianluigi De Gennaro

Saula De Kreutzenberg

Vishwa Deepak

Nikki Delk

Carsten Deppermann

Jeremy P Derrick

Edgar Deu

Kishor Devalaraja‐Narashimha

Vermont P Dia

Cecilia Diaz Oreiro

Dimo Dietrich

Paul Dimayuga

Wei Ding

Francesco Dituri

Giacomo Domenici

Ian Dransfield

Juan Duan

Jerka Dumić

Adrienne Edkins

Aracely Evangelina Chavez‐Pina

Maria Leonor Faleiro

Chao Fang

Candida Fasano

Daniel J Fazakerley

Natalya U Fedosova

Julian Fernandez

Martín Fernández‐Zapico

Rafaela Salgado Ferreira

Roderick Nigel Finn

Eric Allen First

Paul F Fitzpatrick

Louis Flamand

Alison Forhead

Fábio Luis Forti

Salvatore Foti

Marco Fraaije

Agnes François

Katka Franke

Celio G Freire‐de‐Lima

Stephen C Fry

Ying Fu

Naoaki Fujii

Ko Fujimori

Selma P Gago Zachert

Sanjeev Anant Galande

Domenico Galetta

G Ian Gallicano

Tingqing Gan

Vadivel Ganapathy

Bernhard Ganss

Mercedes Garayoa

Armen Yuri Gasparyan

Katja Gehmlich

Pedro MGeraldes

Monika M Golas

Maria Cristina Cintra Gomes‐Marcondes

João Carlos Gomes‐Neto

Weijuan Gong

Dolores Gonzalez Pacanow

Maria Gonzalez‐Siso

Alejandro González‐Torres

Dean Goodman

Koro Gotoh

Ivan T Gout

Lee M Graves

G Grignani

Valentina Grossi

Andreas Gruber

Xiaofei Guan

Sanjeev Gupta

Claudia Hagedorn

F Kent Hamra

Seong Kyu Han

Jennifer Hardingham

Nirmala Hariharan

Larisa M Haupt

Takashi Hayashi

Maoxian He

Jonathan G Heddle

Peter Hemmerich

Karina Herrera Seitz

Kenneth R Hess

Alejandro P Heuck

Eric Hill

Kiichi Hirota

Seiji Hitoshi

Christian Hoischen

Tomoyuki Honda

Cynthia Hong

Jiakai Hou

Hsien‐Da Huang

J Pablo Huidobro‐Toro

Young‐il Hwang

Diego Iglesias‐Gato

Yoshiya Ikawa

Fumiyo Ikeda

Yuri Ikeda‐Matsuo

Hana Im

Marta Irigoyen

Maria Luisa Iruela‐Arispe

Kenichi Ishibashi

Haruto Ishikawa

Hiroshi Ishikita

Takatsugu Ishimoto

Zerrin Isik

Renato Ivanovic

Jyan‐Chyun Jang

Mareike C Janiak

Sabzali Javadov

Thomas Jensen

Marc G Jeschke

Andrew Jheon

Peng Ji

Hongbo Jiang

Nils Johnsson

Nathalie Juge

Susumu Kajiwara

Yasutomi Kamei

Masato Kanazawa

Matheswaran Kandasamy

Koo Jeong Kang

Tomasz Kantyka

Sayako Katada

Takamitsu A Kato

Hibiki Kawamata

Younghoon Kee

Sergio Kellesarian

Istvan Kenessey

Efrat Kessler

Alex Kharitonenkov

Ay Lin Kho

Inna Khozin‐Goldberg

Juliann Kiang

Daisuke Kiga

Jin‐Wook Kim

Hiroyuki Kinouchi

Jason Knight

Sebastian Kobold

Masaaki Komatsu

Vasu Kommireddy

Yutaka Koyama

Katarzyna Koziak

Paweł Kozielewicz

Matthias Kraemer

Oliver H Krämer

Peter Kreuzaler

Susan A Krum

Masato Kubo

Veerapol Kukongviriyapun

Yoshito Kumagai

G La Penna

Sowmya Lakshmi

Jose Maria Landete

Lucia Latella

Jean‐Marc Latour

Yun‐Fai ChrisLau

José Luis Lavín

Maciej Lech

Byung‐Hoon Lee

Hu Lei

Matthias Leippe

Roger Leng

Konstantin Lepikhov

Stephen M Lewis

Klaus Ley

Dongmei Li

Ann Liebert

Chieh‐Yu Lin

Karolina Lisy

Bodu Liu

Andrea Lolli

Cristina Lopez‐Camarillo

David Loria

Hua Lou

Jose Lozano

Huading Lu

Giuseppe Lucarelli

Linlin Luo

Raúl Luque Huertas

Kesem Ma

Miguel Machuqueiro

Mariusz Madej

Mirko Manetti

Francesco Marampon

Silvia Martin

Inés Martín

Inigo Martinez

Enrique Martinez‐Force

Sarabjit S Mastana

Bettie Sue Masters

Rajamma Mathew

Pedro Matias

Konstantinos Mavridis

Kimberly McCall

Peter McKinnon

Dennis Meesters

Jeffery Meier

Frances Meier Gibbons

Gerry Melino

Alexander S Mishin

Yuji Mishina

Yasunori Miyamoto

Mineyuki Mizuguchi

Mario Mondelli

Laura Monturiol‐Gross

Joanna Moraczewska

Masaaki Mori

Hiroyuki Morita

Guilio Muccioli

Daniel Mulvihill

Denis Murphy

Soheil Naderi

Kei Nagashima

Tsutomu Nakamura

Kodanda Nalapa Reddy

Antonio Nanci

Brent Nannenga

David Nebert

Zsófia Nemoda

Shaofa Nie

Aleksandra Nikolic

Takahiro Nitta

Guoping Niu

Takafumi Noma

Marianna Nuti

Edward O'Brien

Delvac Oceandy

Umeharu Ohto

Yohei Okada

Valentyn Oksenych

Fumihiko Okumura

Eugene Boon Beng Ong

Yoshiyuki Otsubo

Fumitaka Oyama

Paula Paccielli Freire

Andres Palencia

Kostas Pantopoulos

Chankyu Park

Derek Parsonage

Christopher Pascoe

Ananya Paul

Sofia Pauleta

Angela Pearson

David S Pederson

Brandt Pence

Xiongbo Peng

Alex Perálvarez‐Márin

Arina Perez

John J Perona

Alexander Pertsemlidis

Alessia Peserico

A Petry

Iva Pichová

Rameshe Pillai

Roxana Pincheira

Indira D Pokkunuri

Alastair W Poole

Richard K Porter

Jan Postberg

Balaji Prakash

Alessandro Prinetti

Bhupesh Prusty

F Pucciani

Amaya Puig‐Kroger

Qiaoping Qin

Jianmin Qiu

Na Qu

Christoph Rademacher

Shofiqur Rahman

Laura Raimondi

Rebecca C Rancourt

Oliver Rando

V Ashutosh Rao

Eduardo Reis

Darren E Richard

Samantha J Richardson

Christophe Richez

Yoshiyuki Rikitake

Jimmy Rincy

Josias Rodrigues

J L Rodrigues‐Fernandes

Carlos Rodriguez

Yueguang Rong

Stefan Rose‐John

Pellegrino Rossi

Tatiana K Rostovtseva

Alex Rothman

A R Rozyanty

Ilaria Russo

Jean‐Marie Ruysschaert

Hiroyuki Saito

Toshiyuki Sakamaki

Hideyuki Sakoda

Joshua Sakon

L Saligan

Paola Sanese

Deborah Schechtman

Gabriele Giacomo Schiattarella

Oliver Schilling

Markus Schosserer

H Steven Seifert

Susana Seixas

Motoaki Seki

Masayuki Sekiguchi

John Shabb

William Shafer

Rahul Sharma

Jie‐hua Shi

Takashi Shichita

Ghizal Siddiqui

N Simoes

Marta Simonatto

Alec Simpson

Juliana Smetana

Ludvig Magnus Sollid

Karly Sourris

Stefania Spanò

Richard Steet

Karin Gunilla Stenkula

Maria Stepanova

Robert Stern

Martin J Stillman

Clelia Storlazzi

James Stowell

Jinsheng Sun

Colleen Sweeney

Erik Swenson

Erin K Sykes

J R Sysol

Katarzyna Szkudelska

Zsuzsa Szondy

Kiyofumi Takabatake

Nobuyuki Takahashi

Takeshi Takarada

Shing Cheng Tan

Bor Luen Tang

Naoyuki Taniguchi

Mahvash Tavassoli

Miguel Sepulveda Teixeira

Vadim Ten

Ugo Testa

Tugsan Tezil

María Tiana

Anne Margaret Tierney

Nestor V Torres Darias

Christina Towers

Michael Tranter

Jason Treberg

Prosum Tribedi

Kangsheng Tu

Pierre Tuffery

Jennifer Tullet

Shusaku Uchida

Yoshihiko Umesono

Tadasu Urashima

Jesús Ureña

Gunes Uzer

Lorea Valcarcel

Willem J H Van Berkel

Raghavan Varadarajan

Stefania Varchetta

Jose‐Luis Vazquez‐Ibar

Giancarlo Vecchio

Adrian Velazquez‐Campoy

A Villagra

Susanne Voelkel

Elmar Wahle

Scott Waldman

Bo Wang

Günther Weber

Jun Wei

Zhengxi Wei

Zhenke Wen

Claudio Chrysostomo Werneck

Kenneth White

Malcolm Whitman

Hans‐Joachim Wieden

Martin Willemoës

Mark A Wilson

Nadine Wiper‐Bergeron

Yongna Xing

Liyan Xu

Meng Xun

Sudhir Kumar Yadav

Hayashi Yamamoto

Atsuko Yamashita

Motohiro Yamauchi

Huihuang Yan

Chin‐Ying Yang

Yao‐Tsung Yeh

S C Yeung

Jason C Young

Di Yu

Nousheen Zaidi

Laura Zannini

Xristo Zarate

Qing‐Yin Zeng

Peng Zhang

Dejian Zhao

Huaiping Zheng

Meng Zhou

Naiqiang Zhu

Anke Zieseniss

